# Potential role of marine algae extract on 3T3-L1 cell proliferation and differentiation: an in vitro approach

**DOI:** 10.1186/s40659-016-0098-z

**Published:** 2016-09-07

**Authors:** Soundharrajan Ilavenil, Da Hye Kim, Mayakrishnan Vijayakumar, Srisesharam Srigopalram, Sang Gun Roh, Mariadhas Valan Arasu, Jong Suk Lee, Ki Choon Choi

**Affiliations:** 1 Grassland and Forage Division, National Institute of Animal Science, RDA, Seonghwan-Eup, Cheonan-Si, Chungnam 330-801 Republic of Korea; 2 The United Graduate School of Agricultural Sciences, Tottori University, Tottori-Shi, 680-8553 Japan; 3 Laboratory of Animal Physiology, Graduate School of Agricultural Science, Tohoku University, Aoba, Sendai Japan; 4 Department of Botany and Microbiology, Addiriyah Chair for Environmental Studies, College of Science, King Saud University, Riyadh, 11451 Saudi Arabia; 5 Bio-center, Gyeonggi Institute of Science and Technology, Suwon, Republic of Korea

**Keywords:** *Chlorella vulgaris*, Chemical constituents, Adipocyte differentiation, PPAR-γ2, AMPK-α

## Abstract

**Background:**

From ancient times, marine algae have emerged as alternative medicine and foods, contains the rich source of natural products like proteins, vitamins, and secondary metabolites, especially *Chlorella vulgaris* (*C. vulgaris*) contains numerous anti-inflammatory, antioxidants and wound healing substances. Type 2 diabetes mellitus is closely associated with adipogenesis and their factors. Hence, we aimed to investigate the chemical constituents and adipogenic modulatory properties of *C. vulgaris* in 3T3-L1 pre-adipocytes.

**Results:**

We analysed chemical constituents in ethanolic extract of *C. vulgaris* (EECV) by LC–MS. Results revealed that the EECV contains few triterpenoids and saponin compounds. Further, the effect of EECV on lipid accumulation along with genes and proteins expressions which are associated with adipogenesis and lipogenesis were evaluated using oil red O staining, qPCR and western blot techniques. The data indicated that that EECV treatment increased differentiation and lipid accumulation in 3T3-L1 cells, which indicates positive regulation of adipogenic and lipogenic activity. These increases were associated with up-regulation of PPAR-γ2, C/EBP-α, adiponectin, FAS, and leptin mRNA and protein expressions. Also, EECV treatments increased the concentration of glycerol releases as compared with control cells. Troglitazone is a PPAR-γ agonist that stimulates the PPAR-γ2, adiponectin, and GLUT-4 expressions. Similarly, EECV treatments significantly upregulated PPAR-γ2, adiponectin, GLUT-4 expressions and glucose utilization. Further, EECV treatment decreased AMPK-α expression as compared with control and metformin treated cells.

**Conclusion:**

The present research findings confirmed that the EECV effectively modulates the lipid accumulation and differentiation in 3T3-L1 cells through AMPK-α mediated signalling pathway.

## Background

Adipogenesis is the process which converts the pre-adipocyte into adipocyte through various mechanisms. In circumstances of positive energy balance, increased energy storage through the conversion of adipocyte from pre-adipocyte by the adipogenesis process [1, 2]. Adipocytes are mainly involved in the maintenance of energy balance and lipid homoeostasis by releasing free fatty acids and storing triacylglycerols in response to changes in energy demands. For this reason, many researchers have been focused on the increase of adipose tissue mass (hyperplasia) and size of adipocytes (hypertrophy) [3]. Animal and human model studies showed that the metabolic disorder of type 2 diabetes mellitus is closely associated with decreased adipogenesis and adipogenic factors [4]. Insufficient amounts of adipose tissue can result in inadequate free fatty acids storage, which leading to increasing plasma levels of free fatty acids, and a resultant increased fat deposition in muscle and liver, which ultimately leads to make insulin resistance and clinical type 2 diabetes [5]. Numerous identified adipogenic factors are involved in the adipogenesis. Among these, peroxisome proliferator—activated receptor-γ2 (PPARγ2), and CCAAT/enhancer binding protein-α (C/EBP-α) plays an important role in adipocyte differentiation and lipid accumulation. Further, the activation of these two factors can stimulate adiponectin, adipogenin, leptin, glucose transporter-4 (GLUT-4), fatty acid synthase (FAS), and adipocyte binding protein (aP2) and other gene expression which are associated with lipogenesis and adipogenesis [6]. AMP-activated protein kinase (AMPK) is a hetero-trimer complex, which acts as a sensor of cellular energy [7]. It activated during stresses that deplete the cellular ATP and phosphorylate the catalytic alpha subunit on Thr172 by an upstream kinase. AMP-activated protein kinase is getting activated during the lipolysis process in adipocyte [8–10].

Natural marine products are much important resources for chemical diversity. Recently, numbers of clinical trials have been conducted on these marine products. From ancient times, marine algae have emerged as an alternative medicine and foods in many Asian countries such as South Korea, Japan and China etc. [11]. *Chlorella* is potent source for protein, lipid soluble vitamins, choline, and dietary fiber. It contains approximately 60 % of protein. The amino acid quantity and nutritional composition of chlorella are similar to egg nutrition’s [12, 13] and it has many biological properties such as promoting growth rate of animals, production of cytokine and stimulating the immune function [14]. Many studies reported that the *Chlorella vulgaris* prevents the oxidative stress in mice and stress mediated ulcer [15]. Further, it has anti-lipidemic and anti-atherosclerotic activity [16]. In addition, *C. vulgaris* prevents dyslipidemia in wistar rats [17]. The present study, we investigated the impact of ethanolic extract of *C. vulgaris* on adipocyte viability and differentiation in 3T3-L1 cells as a model system.

## Results

### Cytotoxicity effect of EECV on 3T3-L1 pre-adipocyte

**Fig. 1:**
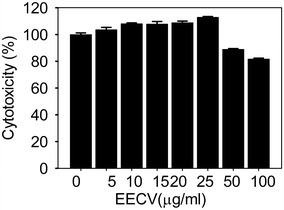
Cytotoxic effects of EECV on 3T3-L1 cells. 3T3-L1 cells were treated with different concentrations (5, 10, 15, 20, 25, 50 and 100 μg/ml) of EECV for 48 h. The concentration at 50 and 100 μg/ml exhibited slight cytotoxic effects on the 3T3-L1 cells

3T3-L1 cells were treated with various concentrations (5, 10, 15, 20, 25, 50 and 100 μg/ml) of EECV for 48 h. EECV treatment did not affect the pre-adipocyte viability up to 25 μg/ml. However, further increment (50 and 100 μg/ml) exhibited slight cytotoxic effects on the 3T3-L1 cells as compared with control. Therefore, we considered safe dosage ranges of EECV were from 5 to 25 μg/ml for further experiment (Fig. 1).

### EECV enhances differentiation and lipid accumulation in adipocyte

**Fig. 2:**
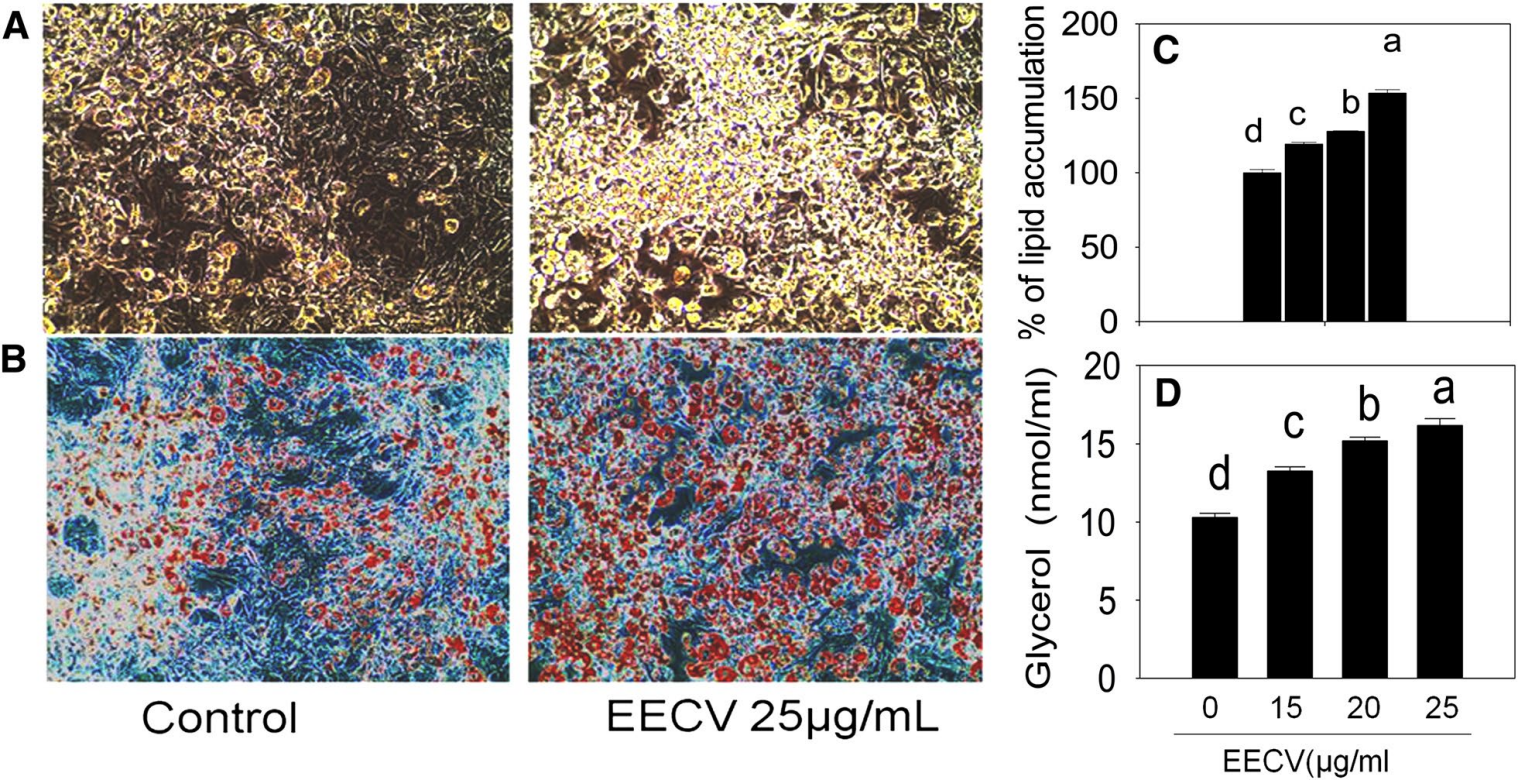
EECV on adipocytes differentiation and glycerol release. **A** Differentiation spots in the experimental adipocytes on the 10th day, **B** oil red O staining of lipids in experimental adipocytes, **C** oil red O stained lipid accumulation extracted by 100 % isopropanol. **D** Glycerol releases from differentiated adipocytes on the10th day. The results represent the mean ± SEM of six replicates. Different letters *a*, *b*, *c*, *d*, within a treatment indicates significant differences (p < 0.05)

Microscopic observation revealed that the 3T3-L1 cells were differentiated into mature adipocyte after addition of differentiation induction medium. This differentiation was enhanced by the EECV treatment (Fig. 2A). The prior concentration 5 and 10 μg/ml of EECV treatment did not influence the adipocyte differentiation. The maximum numbers of mature spherical shape adipocyte were observed at a concentration of 25 μg/ml of EECV. The oil Red O staining results showed greater intracellular lipid accumulation in the cells treated with EECV than the control cells (Fig. 2B). The absorbance of the dye extracted from cells showed higher in EECV treated adipocyte as compared with control adipocyte (Fig. 2C). EECV treated adipocyte showed the higher amount of glycerol releases than the control cells (p ≤ 0.05) (Fig. 2D).

### EECV up-regulates key transcriptional adipogenic and lipogenic gene expression

**Fig. 3:**
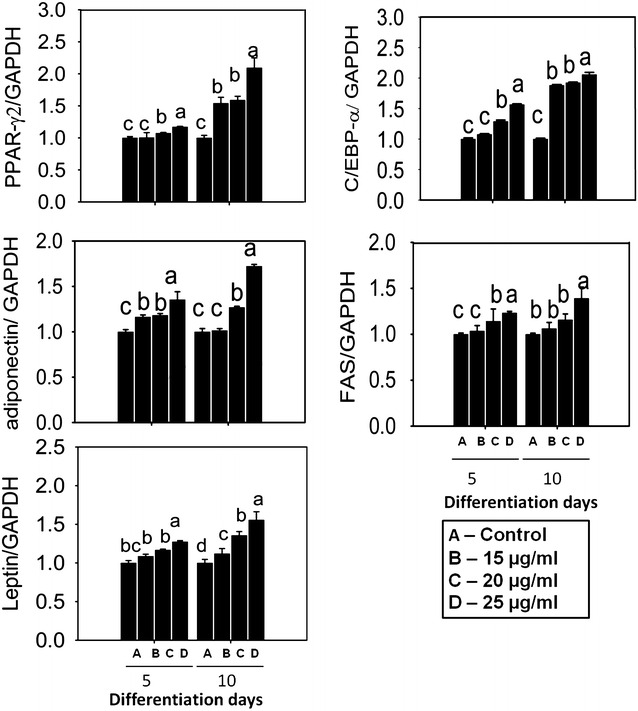
Impact of EECV on adipogenic and lipogenic mRNA expression quantified by qPCR. EECV treatment increased PPAR-γ2, C/EBP-α, adiponectin, FAS and leptin mRNA expression in a dose dependent manner on the 5th and 10th day of differentiation. The results represent the mean ± SEM of six replicates. Different letters *a*, *b*, *c*, *d*, within a treatment indicates significant differences (p < 0.05)

**Fig. 4:**
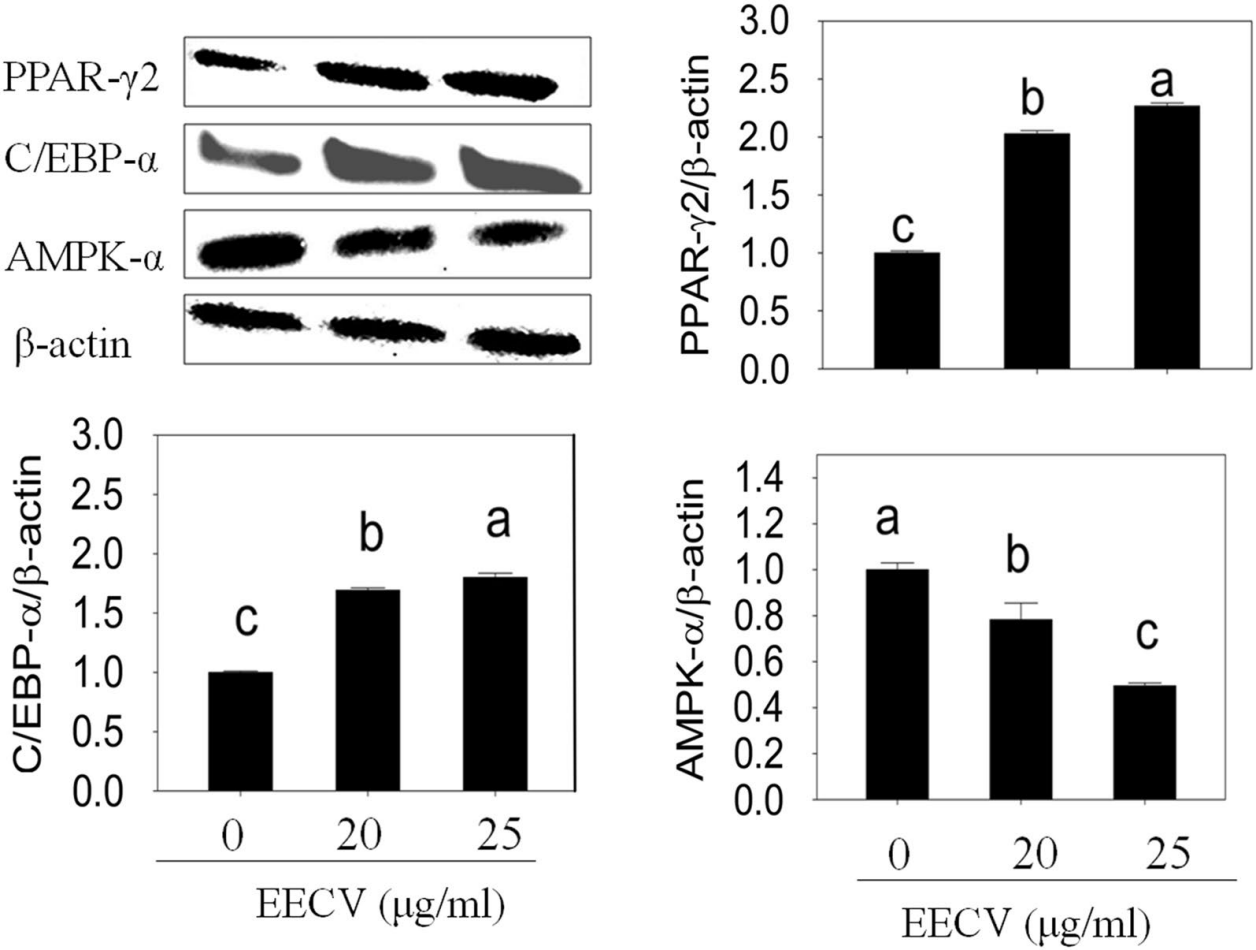
Effects of EECV on PPAR-γ2, C/EBP-α and AMPK-α protein expression. EECV treatment upregulates PPAR-γ2, C/EBP-α and downregulates AMPK-α protein level on the 10th day. The results represent the mean ± SEM of three replicates different letters *a*, *b*, *c*, within a treatment indicates significant differences (p < 0.05)

Further, we investigated the effect of EECV on the adipogenesis-related key transcriptional and other-factors using the qPCR technique. Figure 3 shows, EECV treatment increased PPARγ2, C/EBP-α, adiponectin, FAS, and leptin mRNA expression as compared with control cell. Further, we confirmed the protein expression of PPARγ2 and C/EBP-α by western blot technique. These results evidenced that the EECV upregulates the PPARγ2 and C/EBP-α protein expressions (Fig. 4).

### EECV downregulates AMPK-α activation and upregulates of PPARγ2 protein expression

**Fig. 5:**
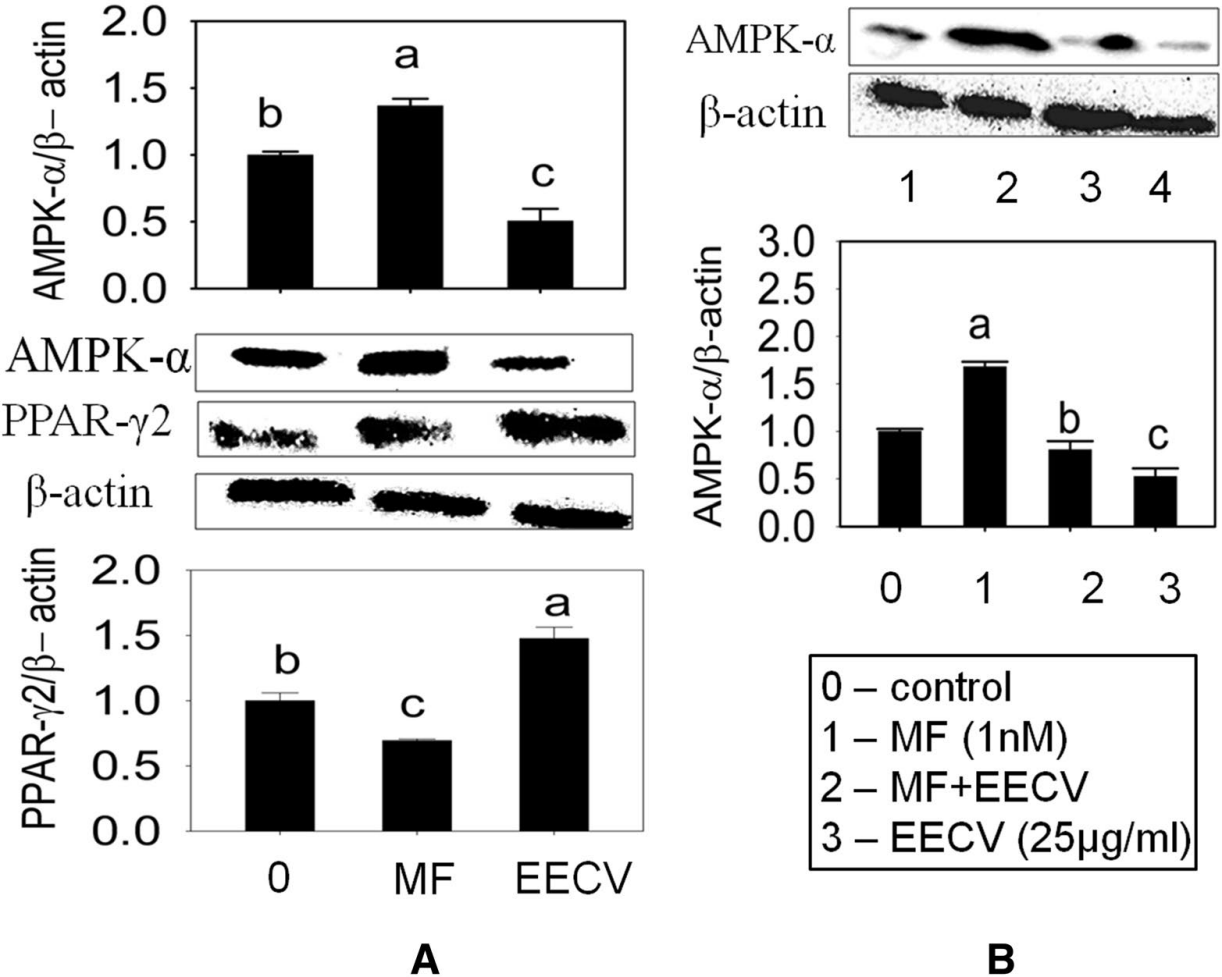
Comparative effects between metformin and EECV on AMPK-α and PPAR-γ2. **A** EECV and metformin alone treated adipocytes exhibited down and upregulation of AMPK-α and PPAR-γ2 respectively. **B** Adipocyte treated with metformin (MF) for 48 h, and then treated with EECV for 8 days. Metformin mediated activation of AMPK-α was disturbed by the EECV treatment. The results represent the mean ± SEM of three replicates different letters *a*, *b*, *c*, *d*, within a treatment indicates significant differences (p < 0.05)

The adipocyte treated with EECV exhibited downregulation of AMP-activated protein kinase (pAMPK) and upregulation of PPARγ2 as compared with control cells. Metformin is an agonist for AMPK, which increased AMPK-α and decreased PPARγ2 expression in adipocyte (Fig. 5A). Further, metformin mediated activation of AMPK-α was abolished by the EECV treatment. It indicates that EECV treatment stimulates adipocyte differentiation via upregulation of PPARγ2 by inhibition of AMPK-α mediated signal pathway (Fig. 5B).

### EECV increased glucose utilization

**Fig. 6:**
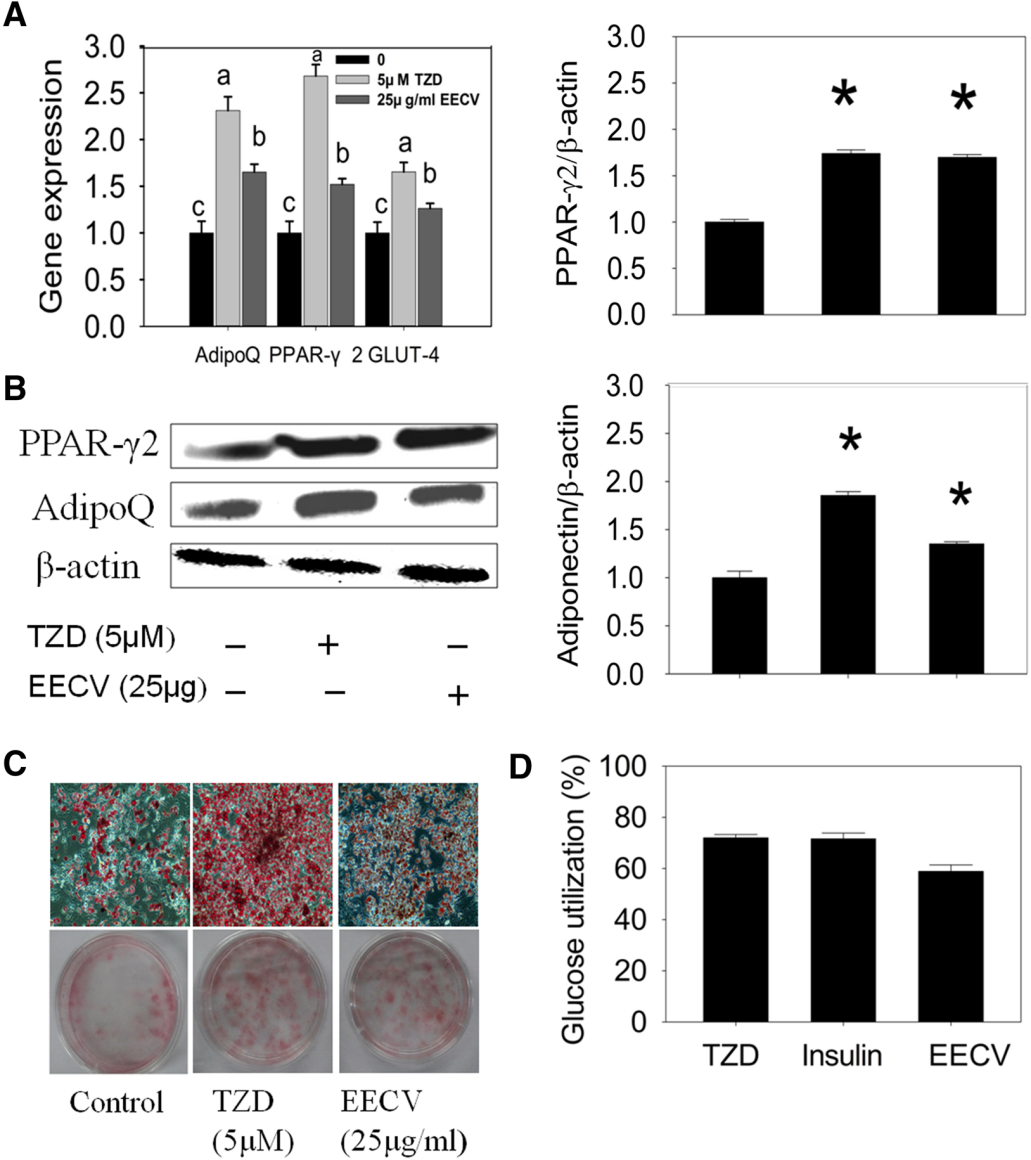
EECV partially mimics like troglitazone. **A** PPAR-γ2, adiponectin (adipoQ) and GLUT-4mRNA expression up regulated by the EECV (25 μg/ml) and troglitazone (TZD) (5 μM). **B** PPAR-γ2 and adiponectin protein expression in control and experimental adipocytes. **C** Lipid accumulation in experimental adipocytes, **D** glucose utilization in differentiated adipocytes by TZD (5 μM), insulin (1 μg/ml), and EECV (25 μg/ml). The results represent the mean ± SEM of six replicates.*p < 0.05 compared to control adipocytes. Different letters *a*, *b*, *c*, within a treatment indicates significant differences (p < 0.05)

Troglitazone (TZD) is an agonist for PPARγ, which stimulates PPARγ expression in the adipocyte. Here, adipocyte treated with troglitazone (5 µM) for 48 h increased PPARγ2 expression and lipid accumulation as compared with control cells. Further, it increased adiponectin and GLUT-4 mRNA expression. Similarly, EECV treatment increased PPARγ, adiponectin and GLUT-4 mRNA expression as compared with control cells (Fig. 6A). Further, we confirmed the protein expression patterns of PPARγ2 and adiponectin in TZD and EECV treated adipocyte; it revealed that the EECV stimulates PPARγ and adiponectin protein expression like TZD treatment (Fig. 6B). EECV stimulates lipid accumulation in adipocyte similar to troglitazone as compared with control cells (Fig. 6C). In addition, adipocyte treated with EECV significantly increased glucose utilisation as compared with control cells. This utilisation may be due to increasing of adiponectin and GLUT-4 level by the EECV treatment. These results were comparable with troglitazone and insulin treatments (Fig. 6D).

### Identification of chemical constituents in extract by LCMS

**Fig. 7:**
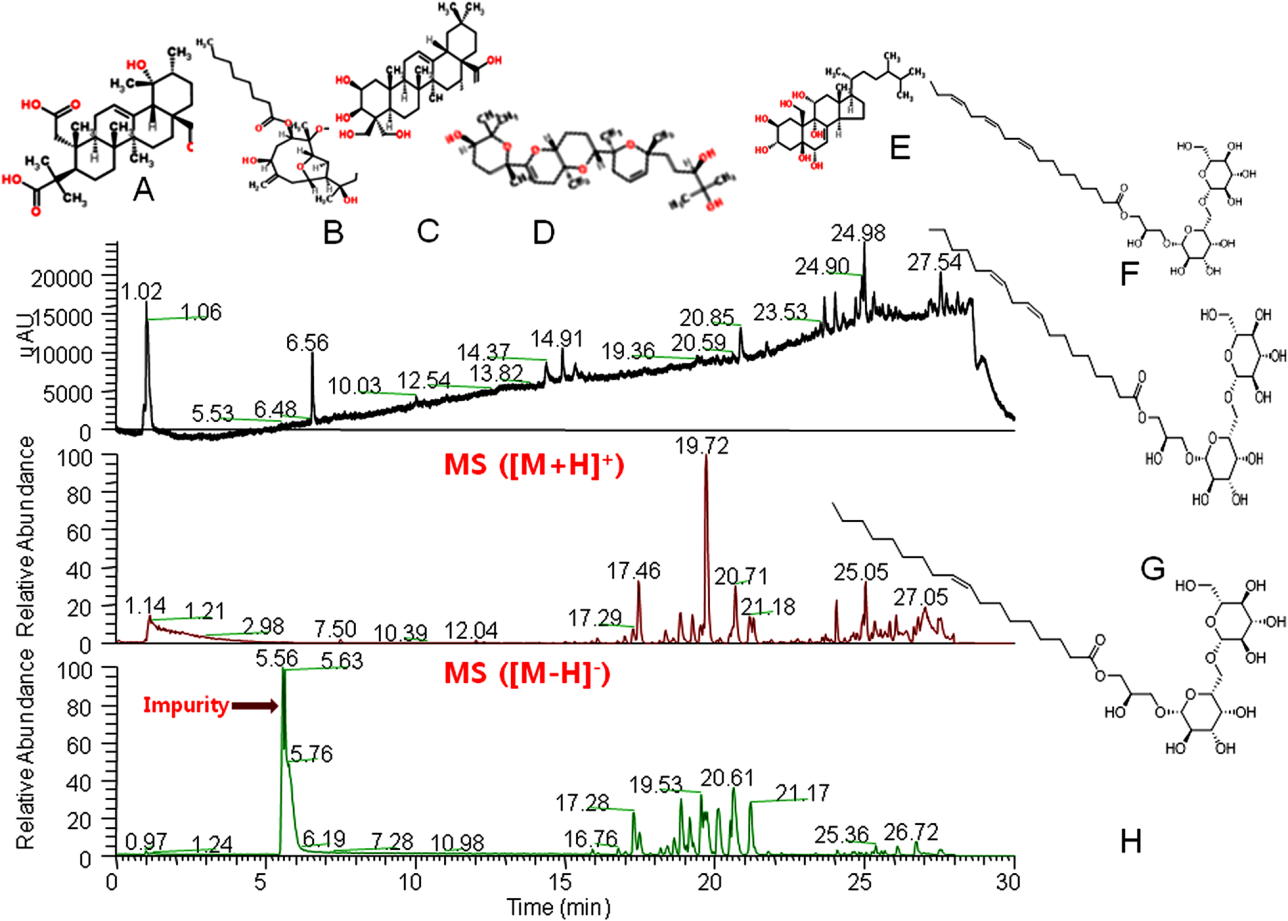
Chemical constituents analysis of extract by LC–MS. *A* Cecropiacic acid, *B* briarellin-A, *C* platycodigenin, *D* martiriol, *E* Ergost-7-ene-2,3,5,6,9,11,19-heptol, *F* ginger glycolipid-A, *G* ginger glycolipid-B, *H* ginger glycolipid-C

The EECV possesses different types of organic compounds including Cecropiacic acid, briarellin-A, Platycodigenin, Martiriol, Ergost-7-ene-2,3,5,6,9,11,19-heptol, Gingerglycolipid-A, gingerglycolipid-B, gingerglycolipid-C (Fig. 7).

## Discussion

Adipocyte differentiation is a process in which pre-adipocyte are developing into mature spherical shape adipocyte which contains more lipids. At the time of adipocyte differentiation, morphological, biochemical characters and lipid accumulation were altered [18].

Natural marine sources have more attraction and attention as pharmacological sources which can be used to develop novel therapeutic agents because of distinct environments of marine life and biological diversity of marine organisms [19]. Many marine compounds possess the different pharmacological application in terms of antibacterial, antidiabetic, anti-inflammatory anti-viral and antifungal activities [20, 21]. In addition, some of the triterpenoid, and saponin has the ability to promotes adipocyte differentiation [22–24]. In the present study, we investigated the ability of the ethanolic extract of *C. vulgaris* to enhance the adipocyte differentiation and lipid accumulation as promising therapeutic agents for dyslipidemia and type-2 diabetes. EECV treatment increased lipid droplets accumulation as compared with control cells which indicate positive regulation of adipogenesis and lipogenesis by the EECV. Further, LC–MS results revealed that EECV had few di, triterpenoids (Cecropiacic acid; Briarellins–A; Martiriol) and saponin (platycodigenin). It might be a reason for accumulation of lipid in adipocytes.

Further, we investigated the impact of EECV on the key transcriptional factors such as PPARγ and C/EBP-α, because of PPARγ and C/EBP-α are much important factors which are working sequentially and co-operatively in stimulating adipogenesis events which lead to accumulation of lipids in the adipocyte [25]. Especially, C/EBP-α has distinct functions in adipocyte differentiation and insulin sensitivity [26]. C/EBP-α plays a major role in the activation and maintenance of PPARγ expression during the adipocyte differentiation. In addition, C/EBP-α stimulates the gene expressions which are involved in the insulin sensitivity, lipogenesis, lipolysis and another encoding gene [27, 28]. Our research findings indicates that the increased expression of PPARγ2 stimulate the adipocyte differentiation continuously in EECV treated adipocyte by direct acting on C/EBP-α. Similarly, extract from *Italian rye grass* [29] and *Mulberry* leaf [30] stimulates the adipocyte differentiation through activation of PPARγ and C/EBP-α.

Adiponectin is an important hormone that synthesized and expressed exclusively in the differentiated adipocyte. Adiponectin level was increased in the fully differentiated adipocyte. It regulates numbers of metabolic processes either by enhancing insulin sensitivity in muscle and liver or by stimulating fatty acid oxidation in many tissues [31–33]. Over-expression of adiponectin enhances insulin sensitivity in part through hepatic glucose production [34]. Increase of adiponectin mRNA expression in the adipocyte is one of the main criteria for lipid accumulation in the adipocyte. Our finding strongly supports the above said suggestion, in that EECV treatment increased lipid droplet and glycerol accumulation in adipocyte may be due to increasing of adiponectin.

Leptin is a peptide hormone which is exclusively secreted in adipocyte and it has many biological activities such as effects on appetite, food intake, body weight regulation, fertility, reproduction and hematopoiesis [35]. Leptin mRNA is stimulated during adipocyte conversions, which are useful for studying adipocyte differentiation and their function under controlled conditions [36]. The present study, adipocyte supplemented with different concentration of EECV upregulates leptin mRNA expression. This result was strongly concurred with lipid accumulation report, which reveals EECV treatments increased lipid accumulation, because leptin enhance the lipid accumulation in adipocyte [37].

Fatty acid synthase (FAS) a multi-enzyme protein, plays a vital role in the anabolic conversion of dietary carbohydrates to fatty acids and regulation of lipid metabolism [32, 38]. Fatty acid synthase promotes the synthesis of triglyceride and cytoplasmic accumulation in the adipocyte [39]. The expression of PPARγ and C/EBP-α are much important for activation of other adipocyte specific genes and adipokines such as FAS, leptin, and adiponectin [40, 41]. Among these, fatty acid synthase is a lipogenic enzyme which involves in the fatty acid synthesis and it’s largely upregulated in the cells or tissues [42]. The 3T3-L1 pre-adipocyte are differentiated with IBMX and dexamethasone and acquire the characteristic of fat cells including responsiveness to insulin and induction of FAS [43]. The present study, we demonstrated that 3T3-L1 cell treated with different concentration of EECV increased expression of FAS mRNA as compared with control cells. This result suggested that the EECV has lipogenic activity by activating FAS mRNA expression.

The AMP-activated protein kinase (AMPK) plays an important role in metabolic energy homeostasis in cells. When it activated, it switches on the catabolic pathways and simultaneously switches off the ATP consuming anabolic pathways [44]. AMPK-α provides an upstream signal of peroxisome proliferator-activated receptor γ (PPAR γ), which inhibits the adipocyte differentiation [45, 46]. In addition, AMPK directly modulates fatty acid synthesis and oxidation by changing expression patterns of proteins and enzymes, which involved in the fat metabolism and also AMPK-α regulates pre-adipocyte differentiation [41, 47]. Many reports claimed that the AMPK-α pathway is responsible for the inhibition of adipocyte differentiation by several natural compounds like apigenin dioxinodehydroeckol, Chitin, Ginsenoside, epigallocatechin gallate, 5-Aminoimidazole-4-carboxamide-1-β-D-ribo-furanoside (AICAR) [48–53]. The present study, EECV decreased the AMPK-α and increased expression of PPAR γ2 during differentiation, whereas addition of metformin is an agonist for AMPK-α exhibited downregulation of PPAR γ2 in the adipocyte. Further, metformin mediated AMPK-activation in adipocyte was inhibited by EECV treatment. It indicates that EECV increased the adipocyte differentiation and lipid accumulation via up-regulation of PPAR γ2 by the inhibition AMPK-α expression.

Thiazolidinediones (TZDs) is types of insulin sensitizing drugs used for type 2 diabetes treatments and it ameliorate insulin resistance by promoting the adipocyte proliferation and differentiation through up-regulation of PPAR-γ in the adipocyte. Further, it stimulates the adiponectin secretion in obesity animal model and humans [54, 55] which activating the gene responsible for adipocyte differentiation.27 muscle and adipose tissue possess the two kinds of glucose transporter such as GLUT-1 and GLUT-4 in which GLUT-4 is an important insulin regulator [56]. Down-regulation of glucose uptake in muscle and adipose tissue is an important reason for insulin resistance in type2 diabetes mellitus patients [57]. The present study, troglitazone treatment stimulates the adiponectin, PPAR-γ, and GLUT-4 mRNA expression in the adipocyte. We found almost similar magnitude responses in EECV treatment; it stimulates the adiponectin, PPAR-γ, and GLUT-4 expression. Further, the EECV enhances the glucose utilization in adipocyte through up-regulation of adiponectin and GLUT-4 expression. Generally, GLUT-4 involved in transporting of glucose into adipose tissue and muscle [58]. These data suggested that EECV increased glucose utilization in the adipose tissues via activation of adiponectin and GLUT-4 expression.

## Conclusions

Type-2 diabetes mellitus is closely associated with adipocyte differentiation. Insufficient adipocyte differentiation is one of the main reasons for developing type-2 diabetes. Any deficiency in the adipocyte differentiation causes fat depositions in the liver and muscle which are making insulin resistance and type-2 diabetes. The data presented in this study indicate that *C. vulgaris* effectively activate the adipocyte differentiation and glucose utilization. Further, we identified some of the triterpenoids and saponin compounds in this extract, which may be responsible for the positive regulation of adipogenesis. Hence, the present findings suggested that the EECV effectively modulates the lipid accumulation and differentiation in 3T3-L1 cells through AMPK-α mediated signalling pathway. Further, investigation needs to determine its active adipogenic molecule present in the *C. vulgaris.*

## Methods

### Chemicals and cell

The 3T3-L1 preadipocyte was obtained from the American Type Culture Collection (Rockwille, MD, USA). Dulbecco modified Eagle medium (DMEM) and fetal bovine serum was procured from Gibco-BRL (Gaithersburg, MD, USA). The EZ-Cytox kit, mRNA extraction and RT-PCR kits were purchased from Invitrogen (iTSBiO. Seoul, South Korea and Carlsbad, CA, USA). IBMX, Dexamethasone, Insulin, troglitazone, oil red O stain, isopropanol and formalin were purchased from sigma Aldrich, USA).

### Extract preparation

The *C. vulgaris* powder purchased from Daesang, Seoul, South Korea and it was soaked in 70 % ethanol for 72 h at 37 °C by intermittent mixing using an orbital shaker. After that, the mixture was filtered using Whatman filter paper to separate the supernatant and the sediment. The filtrate was concentrated under reduced pressure at 40 °C until extraction solvent was completely removed, stored in refrigerator at 4 °C for further experiments.

### Cytotoxicity assay

The water soluble tetrazolium [WST; 2[2-Methoxy-4-nitrophenyl]-3[4-Nitrophenyl]-5-[2, 4-disulfophenyl]-2-H-tetrazolium monosodium salt (EZ-Cytox kit) was used for analysis of cytotoxicity. The cells were seeded in the 96 well at a density of 1 × 10^4^ cells/well. After, 24 h, cells were exposed to the different concentration of EECV 5, 10, 15, 20, 25, 50 and 100 µg/ml]. It was incubated at the 37 °C in 5 % CO_2_ incubator for 48 h and then the culture was treated with WST incubated for 2 h. Then, the intensity of colour was measured at 450 nm using spectra count ELISA reader (Packard Instrument Co., Downers Grove, IL, USA).

### Cell culture and experiment

Adipocyte differentiation experiment was carried out by the method of Choi et al. [59] with a slight modification. Briefly, 3T3-L1 cells were seeded in 12 well culture plates at a density of 1.5 × 10^4^ cells/well. Cells were incubated at 37 °C with 5 % CO_2_ and culture medium was replaced by every 48 h. After 2 days of 100 % confluence of 3T3-L1 cells, the growth media was replaced by differentiation media (DMEM containing 10 % FBS, 0.5 mM 3-isobutyl-1-methylxanthine, 1 μM dexamethasone, and 1.7 μM insulin) for 48 h. The pre-adipocyte were maintained and re-fed every 48 h with 10 % FBS-DMEM medium. To examine the effect of EECV on adipocyte differentiation, the pre adipocyte received 5, 10, 15, 20 and 25 µg/ml EECV (in DMEM medium) every 2 days started at 2 days post confluence until the end of the experiment days.

### Quantification of lipid content by oil red O staining method

3T3-L1 cells were fixed with 10 % formalin for 1 h. The cells were then rinsed with 40 % isopropanol and 3 ml of oil red O staining solution was added to each well, and incubated at room temperature for 15 min and then washed thrice with distilled water and photographed by an inverted microscope (CKX41, Olympus Corporation, Tokyo Japan). Additionally, oil red O stain was eluted from the adipocyte using isopropanol (100 %) and measured at 490 nm [59].

### Measurement of glycerol accumulation

According to adipolysis assay Kit procedure (CHEMICONH International Inc. Temecula, CA, USA), the glycerol level was quantified. In detail, differentiated adipocyte in the presence of different concentration of EECV was washed twice with PBS (Gibco, Thermofisher scientific, Seoul, South Korea) and further incubated with lipolysis buffer for 1 h. Then, the buffer was collected and measured the glycerol concentration using microplate reader at 540 nm. The amount of glycerol was calculated using the standard curve of glycerol.

### Glucose utilization

The differentiated adipocyte was exposed to high glucose (25 mM) containing DMEM medium with EECV (25 μg/ml), insulin (1 μg/ml) and troglitazone (5 μM), individually for 24 h. An assay, without EECV was considered as control. After 24 h, the concentration of glucose in spent medium was estimated with a commercial assay kit (Sigma Aldrich-GAGO-20).

### Quantification of adipogenic gene expression using quantitative RT-PCR

Cellular RNA was isolated from 3T3-L1 cells using the RNeasy lipid tissue mini kit (Qiagen, Valencia, CA, USA) according to the manufacture instructions. Isolated cellular RNA was measured by UVS-99 micro volume UV/Vis spectrometer-ACT gene (ACTGene, Piscataway, NJ, USA). Five hundred nanogram of the cellular RNA was used for cDNA synthesis with oligo primer (dT) and reverse transcriptase (superscript III first strand synthesis system for RT-PCR)-Invitrogen. Real-time PCR was conducted using an ABI 7500 PCR system (Applied Biosystem, Foster City, CA, USA). The levels of target gene cDNA were measured by SYBR green-based real time PCR in 10 μl of 5 μl Power SYBR green-based mix reaction buffer (Biorad, Seoul, South Korea), 10 pΜ of forward (F) reverse (R) primers, 1 μL cDNA and 3 μL DEPC water. All the expression levels were normalized to the housekeeping gene.

#### Protein extraction and immune blot analysis

Proteins from experimental adipocyte were extracted by RIPA lysis buffer with 1X protease inhibitor cocktail (Roche, Basel, Switzerland). The cells were washed with PBS for thrice. Then, added 500 μl RIPA lysis buffer and incubated at 4 °C for five min. After that, cells were scraped rapidly with a cells scrapper to remove and lyse residual cells and transferred cell lysate to a tube for centrifugation at 4 °C for 10 min. The total protein concentration was measured by a Bio-Rad protein assay kit. An equal amount of protein samples were separated by SDS-PAGE (10 %) and trans blotted onto polyvinylidene difluoride (PVDF) membranes (iBlot gel transfer stacks, Novex, Life Technology, Waltham, MA, USA). According to western breeze chemiluminescence protocol (Invitrogen, USA), the immunobloting was performed (except primary antibody incubation time and temperature) with rabbit monoclonal antibody of specific proteins such as PPRAγ2, C/EBP-α, adiponectin, AMPK-α and β-actin (Cell Signaling Technology, Danvers, MA, USA). The signals were observed with an enhanced chemiluminescence kit (Biorad, Seoul, South Korea) by a chemiluminescence imaging system (Davinch Chemiluminescence, Seoul, South Korea) and the intensity of immune reacted bands was quantified by the ImageJ software-1.49 version (32 bit), Wayne Rasband, National Institute of Health, USA.

#### LC–MS/MS analysis

The chemical constituents of EECV were analyzed by An API 4000 Q TRAP tandem mass spectrometer (Applied Biosystems, Foster City, CA, USA), equipped with an Agilent 1200 series HPLC system (Agilent Technologies) and with an electrospray ionization tandem mass spectrometry (ESI–MS/MS) source in positive ion mode [[M + H]^+^]. The analytical conditions are: start (100 amu), stop (1300 amu), and scan time (4.8 s); curtain gas, 20 psi (N2); heating gas temperature, 550 °C; nebulising gas, 50 psi; heating gas, 50 psi; ion spray voltage, 5500 V; declustering potential, 100 V; entrance potential, 10 V.

### Statistical analysis

Each experiment was carried out in replicates (n = 6; n = 3). Data are expressed as mean and standard error of mean (SEM). Statistical analysis was carried out using, Excel and one way ANOVAs and multivariate comparisons using the statistical package of social science (SPSS) program [Version 16.0] (SPSS, Inc., Chicago, IL, USA) and significance were represented as p < 0.05.
